# “One Can Have All Sorts of Good Intentions but Sometimes in Everyday Life, It Turns Out Differently”: Change Agreements in Couples' Everyday Lives

**DOI:** 10.1111/jmft.70179

**Published:** 2026-09-29

**Authors:** Nanna Fensman Lassen, Hanne Nørr Fentz, Klaus Nielsen, Tea Trillingsgaard

**Affiliations:** ^1^ Department of Psychology and Behavioral Sciences The Couple and Family Research Unit, Developmental Psychology, Aarhus University Aarhus C Denmark; ^2^ Department of Psychology and Behavioral Sciences Research Unit for Practice‐Based Learning and Centre for Qualitative Method Development, Aarhus University Aarhus C Denmark

**Keywords:** change processes, longitudinal interpretative phenomenological analysis, longitudinal qualitative study, online couple intervention, ourrelationship, relationship distress

## Abstract

Online couple interventions are increasingly used to support couples experiencing relationship distress. However, little is known about how couples integrate intervention elements into their everyday lives. This longitudinal qualitative study examined how distressed parenting couples integrate change agreements developed in the online OurRelationship program. Six couples (12 participants) were included in the study. Data consisted of one coach session, as well as two rounds of individual interviews conducted 3–6 weeks and 6–7 months after program completion. Using longitudinal interpretative phenomenological analysis, we identified three main ways couples experienced, interpreted, and used the change agreements: (1) as instrumental contracts, (2) as expressions of mutual understanding, and (3) as making relational responsibility visible, including potential gendered asymmetries. Findings suggest that change agreements do not transfer directly into couples' everyday lives and that consequences evolve over time. Implications for practitioners, intervention design, and theories of relational change are discussed.

## 1 Introduction

In recent years, the field of couple therapy has witnessed a substantial increase in the development and dissemination of online couple interventions (Bradbury and Bodenmann 2020). These interventions have emerged partly in response to a well‐documented gap between the large number of couples experiencing relationship distress and the relatively small proportion who seek or receive traditional in‐person couple therapy (Doss et al. 2003; Johnson et al. 2002; Trillingsgaard et al. 2019). Online interventions such as OurRelationship (OR), Power of Two, ePREP, and Hold Me Tight provide accessible, flexible, and cost‐effective alternatives to traditional couples therapy (Doss et al. 2022). Such programs may be particularly attractive and important options to couples who face financial, logistical, or psychological barriers to attending couple therapy, including social stigma, scheduling constraints, high costs, concerns about trustworthiness of the therapist, or limited availability of trained and qualified clinicians (Hubbard and Anderson 2022).

A growing body of research indicates that online couple interventions can support improvements in relationship satisfaction and functioning (Carr 2025). This paper focuses on the 8‐week online OR program, which is based on Integrative Behavioral Couple Therapy (IBCT), and is currently the most extensively studied online self‐help program for couples (Doss et al. 2023). OR was developed as a secondary intervention: it is designed to help couples experiencing relationship distress, but before those problems escalate and necessitate the involvement of a couple therapist (Doss et al. 2013). This distinction helps situate OR within the broader continuum between relationship education and therapeutic intervention (Myers‐Walls et al. 2011).

US‐based randomized controlled trials conducted by the program's developers demonstrate sustained improvements in self‐reported relationship satisfaction, reduced levels of couple conflict, and improvements in participants' mental and physical health (Doss et al. 2016; Doss et al. 2019; Roddy et al. 2021; Roddy et al. 2020). The positive effects on relationship satisfaction and communication conflict have been replicated in cross‐cultural samples (Fentz et al. 2025; Trillingsgaard, Hansen, et al. 2025). Additionally, benefits have been found for children's emotional and behavioral outcomes (Doss et al. 2020) and across vulnerable populations, including low‐income couples, ethnic minority groups, and veterans (Doss et al. 2020; Georgia Salivar et al. 2020; Georgia Salivar et al. 2018; Hatch et al. 2022; Le et al. 2020; Mitchell et al. 2023).

However, most studies focus on treatment efficacy, offering limited insight into how couples integrate intervention elements into everyday life, particularly from a longitudinal perspective. Understanding how intervention elements are experienced, interpreted, and used within the ongoing practices of couple relationships is an important yet underexplored dimension of couple intervention research. This is crucial, as it is in everyday life that such changes can and must make a meaningful difference. The present study aims to address this gap by focusing specifically on couples' change agreements (CAs) developed in the final module of the online program OR.

### 1.1 Contextualizing Change Agreements Within the Field of Behavioral Couple Therapy

CAs are specific, actionable commitments that partners initially formulate individually through their OR program activities. The CAs are subsequently discussed and agreed upon in a structured partner‐to‐partner conversation to guide behavioral changes in communication, reconciliation, and the core concerns couples choose to address within OR (Doss et al. 2013). Common examples of CAs include requesting time‐outs during conflicts, engaging in structured speaker‐listener conversations, expressing previously unspoken emotions, initiating reconciliation following an argument, and planning regular date nights.

Within OR, these CAs are formulated after couples have worked through the *Observe* and *Understand* modules. The Observe and Understand modules guide couples in identifying one or two core concern(s) in their relationship and developing a deeper and more nuanced understanding of the relational patterns that maintain them. These modules primarily emphasize emotional acceptance and mutual understanding (Doss et al. 2013). The third module, *Respond*, shifts toward specific behavioral actions and focuses on solutions and positive change, thereby aiming to support couples in translating insights from the two first modules into concrete behavioral changes (Doss et al. 2013).

Within the broader history of behavioral couple therapy, explicit behavior‐change strategies have long played a central role. Traditional Behavioral Couple Therapy (TBCT) emphasize direct modification of partners' negative or undesirable behaviors through traditional behavioral techniques such as communication training, problem‐solving, and behavioral activation (Jacobson and Margolin 1979). These strategies sought to improve relationship functioning by increasing positive exchanges and reducing negative patterns of interaction through mutually agreed‐upon behavioral changes.

According to more recent and contextually oriented behavior theory (Christensen et al. 2020) such strategies rely on rule‐governed principles for behavior change (Skinner 1966), in which individuals regulate their actions by following rules, prescriptions, and agreements about how they should behave in particular situations. Although TBCT historically have demonstrated substantial efficacy (Hahlweg and Markman 1988), questions emerged regarding the durability and generalization of behavior changes that rely primarily on adherence to explicit rules. For example, while a meta‐analysis reported a large overall effect size for TBCT (*d* = 0.95) (Hahlweg and Markman 1988), rates of clinically significant change have been more modest: 54.7% of couples experienced reliable improvement post‐treatment, and 35.3% achieved both reliable improvement and moved into the nondistressed range (Jacobson et al. 1984). Moreover, studies suggest that treatment gains may diminish over time, with mixed long‐term outcomes (Christensen et al. 2006; Hahlweg and Markman 1988; Jacobson et al. 1984). In the emotionally complex and situationally variable contexts of everyday couple life, rule‐governed behavioral prescriptions may be difficult to sustain consistently (Christensen et al. 2020).

IBCT was developed partly in response to these concerns (Jacobson and Christensen 1996). Unlike traditional behavioral or cognitive couple therapies, IBCT emphasizes emotional acceptance and mutual understanding between partners as primary goals over cognitive restructuring or behavioral change. However, traditional behavioral interventions and tolerance‐building may be introduced as secondary strategies once sufficient acceptance between partners has been established (Christensen et al. 2020).

Thus, IBCT places greater emphasis on contingency‐shaped change (Christensen et al. 2020; Skinner 1966). From this perspective, new patterns of interaction are assumed to emerge gradually as partners develop a deeper understanding of each other and continue to respond to each other's behaviors, rather than by adhering to behavioral rules or prescriptions for specific actions in particular situations. One randomized clinical trial suggests that while TBCT may help couples with relatively rapid early improvements in couple satisfaction, these improvements seem to plateau later in treatment. In contrast, couples in IBCT may experience improvements slower but more gradually across treatment, with gains that reach the same level as TBCT but remain more stable over time (Christensen et al. 2004; Christensen et al. 2006).

OR is based on IBCT principles (Doss et al. 2013), particularly its emphasis on facilitating partners' understanding of patterns of often negative or destructive interactions and fostering empathic responses and acceptance between partners (Doss et al. 2016). At the same time, the program incorporates structured elements designed to help couples translate such insights into concrete behavioral changes (Doss et al. 2013). The Respond module exemplifies this integration by inviting couples to formulate explicit CAs about how they will approach difficult situations or destructive patterns of interactions in their relationship moving forward.

This raises important questions about how such CAs are experienced, interpreted, and used by couples within their everyday lives over time. On the one hand, collaboratively formulated agreements may help partners translate new understandings into actionable relational practices. On the other hand, explicit agreements may also invite a more rule‐governed orientation to relational change in which partners monitor, evaluate, or attempt to enforce adherence to agreed‐upon behaviors.

One prior study suggests that the Respond module may play an important role in sustaining program effects (Roddy et al. 2017). In their evaluation of a brief version of OR that excluded the Respond module, and thus the CAs, Roddy et al. (2017) found that although couples initially showed improvement, these gains had largely dissipated by the 3‐month follow‐up. The authors proposed that omitting the Respond module and the CAs may have limited couples' ability to translate insights from the program into concrete changes in their everyday lives. However, the reduced time available for couples to focus on their relationship concerns, develop greater mutual understanding, and practice more empathetic responses may also have contributed to these outcomes. Consequently, the processes driving the findings remain unclear, and further in‐depth examination is needed before any conclusions can be drawn.

### 1.2 An Everyday Life Perspective Informed by Critical Psychology

To explore how partners experience, interpret, and use the CAs developed in the Respond module, the present study is theoretically inspired by critical psychology (Dreier 2008; Holzkamp 1998). From this perspective, therapeutic interventions are not understood as isolated events confined to sessions or program engagement (Mackrill 2008). Rather, their meanings and consequences are constituted in relation to partners' conduct of everyday life. The concept of conduct of everyday life refers to the ongoing activity through which partners organize, integrate, and construct their daily lives “each and every day,” coordinating and managing the various—and sometimes contradictory—demands they encounter while simultaneously expanding their possibilities for action (Holzkamp 1998).

This perspective emphasizes that everyday life is not something that simply happens to partners. On the contrary, partners are seen as situated subjects who actively participate in and shape various social practices from a first‐person, situated stance. In this way participation in an online self‐help program is not simply what happens during the online sessions or within the program. Rather, the significance is constituted in the broader everyday life contexts in which partners live outside their participation in the program.

Participation is a key concept within this theoretical framework. It means that we “think of human subjects as already involved in social practice of which we have a more or less common understanding” (Dreier 2008, 30). As Dreier (2015, 127) points out, “to intervene” means “to come, be or lie between” and “to come between as an influence, as in order to modify, settle, or hinder some action, argument, etc.” Thus, to intervene as a therapist, a coach, or through an online intervention is to become a potential influence among many other things that are already taking place in the partners’ conduct of everyday life.

### 1.3 The Current Study

This perspective on interventions and everyday life motivates the present study, which is guided by the following research questions:

1. How do partners experience, interpret, and use the CAs they agree on in the Respond module of OR within their everyday lives?
2. What types of consequences do the different ways of experiencing, interpreting, and using the CAs have over time?

By examining how partners experience, interpret, and use the CAs beyond the structured context of the intervention, the study aims to contribute to an enhanced understanding of how relational change efforts initiated within a structured online couple intervention are sustained, reshaped, or challenged in the ongoing conduct of everyday life.

## 2 Method

The present study is a longitudinal qualitative study aimed at gaining an in‐depth understanding of couples' experiences, interpretations, and use of CAs. Data consists of semi‐structured individual interviews with each of the 12 participants who completed the OR program. Individual interviews were chosen to enable participants to articulate distress, ambivalence, or critical perspectives that they might otherwise withhold in the presence of their partner (Cerchiaro 2023; Throsby and Gill 2004; Zarhin 2018).

Participants were invited to take part in two interviews approximately 3‐6 weeks (T2) and 6‐7 months (T3) after completing OR. In addition to these interviews, data from five coach sessions conducted as part of the OR program for each couple also informed the analysis. The fifth coach session (T1) was analyzed in depth because it focused on the couples’ formulation of CAs, while the remaining sessions provided contextual material. An overview of the study design is presented in Figure 1.

**Figure 1:**
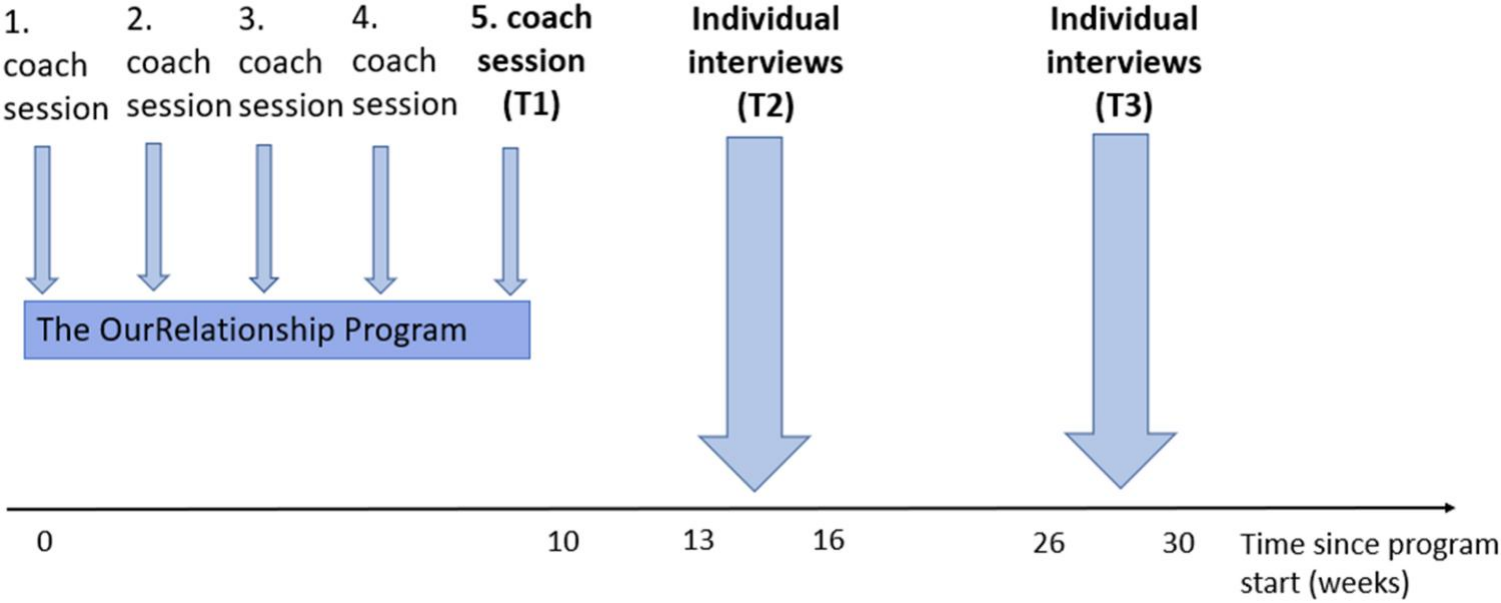
Study design. T1 = time point 1 (fifth coach session); T2 = time point 2 (individual interviews conducted 3–6 weeks after program completion); T3 = time point 3 (individual interviews conducted 6–7 months after program completion); OR = OurRelationship (Doss et al. 2013). [Color figure can be viewed at wileyonlinelibrary.com]

In the article T1 refers to the fifth coach session, T2 to the first individual interview, and T3 to the last individual interview with the participant.

### 2.1 Participants

Participants were recruited from a larger three‐arm randomized controlled trial investigating self‐help for parenting couples (Trillingsgaard, Fentz, et al. 2025; Trillingsgaard et al. 2026). Following an initial assessment, eligible couples were randomized to OR, bibliotherapy (BibT), or waitlist. After the waitlist period, couples in the waitlist condition chose between OR and BibT. Of the 374 couples in the larger three‐arm RCT, 111 were allocated to the waitlist, of whom 90 subsequently initiated an intervention and made a choice; 77 selected OR (70 with coaching and 7 without), and 13 selected BibT (seven with coaching and six without). The 65 couples who completed OR with coaching constituted the recruitment pool for the present qualitative study.

Given the idiographic and longitudinal design, we aimed to include five couples to enable in‐depth examination of individual and couple trajectories, with one additional couple recruited as a buffer against potential attrition. Of the 13 couples approached, six (46.2%) agreed to participate and seven (53.8%) declined. The six participating couples (12 participants) provided data at three planned time points, enabling longitudinal analysis at the individual, couple, and cross‐couple levels while retaining an in‐depth idiographic focus (Smith and Osborn 2015).

Couples were initially assessed and found eligible, if they met at least one of the following criteria: (1) One or both partners reported at least one act of physical or psychological aggression during conflicts in the past 3 months. (2) One partner scored at least 1 standard deviation (SD) (indicating severe low levels of relationship satisfaction) or both partners 0.5 SD below the Danish population mean on relationship satisfaction (measured by the Couple Satisfaction Index, CSI‐4; see Leth‐Nissen et al. 2024 for Danish norms).

Additional eligibility requirements included: both partners being 18 years or older, currently cohabiting for at least 6 months, having at least one child under the age of 18 living at home, being able to read and understand Danish, and having access to the internet, a tablet, computer, or smartphone. Couples were excluded if one or both partners reported ongoing divorce or separation, were engaged in an affair, or screened above threshold for suicide risk. Couples were also excluded if acts of physical aggression resulted in either injury or fear within the past 3 months.

Six different‐sex couples participated; five were married. Women were aged 32–50 years (*M* = 40.50) and men 34–51 years (*M* = 41.83). Couples had 1–3 children (*M* = 2) and lived in four municipalities in Denmark. All participants were White/Caucasian and had completed compulsory schooling. Educational attainment ranged from vocational to long‐cycle higher education, and 11 of 12 participants were employed.

All participants took part in the coach sessions. Five couples completed both individual interviews; one couple (Sarah and Michael) did not participate in the follow‐up interview. Following OR, three couples were offered couple therapy as part of the stepped‐care model. Sarah and Michael declined the offer, whereas Fred and Maya and Jane and John accepted it. None had begun couple therapy at the time of the interviews.

### 2.2 Procedure

All interviews were conducted by the first author, who had no prior coaching, recruitment, or screening involvement with the participants. This separation of roles was intended to support openness for the participants. T2 interviews were conducted between October 30, 2024, and July 2, 2025, and T3 interviews between October 2025 and January 2026. All interviews were conducted online via Zoom and lasted 31‐77 min.

Separate interview guides for T2 and T3 covered participants' experiences and interpretations of OR and CAs, their use in everyday life, perceived changes and challenges, and the meanings and consequences attributed to CAs. Interviews were conducted as flexible, exploratory dialogs, allowing the interviewer to pursue issues raised by participants. They were recorded and transcribed verbatim. Quotations have been translated from Danish into English to preserve the meaning and communicative intent of the participants' statements.

### 2.3 Reflexivity

It should be noted that N.F.L., H.N.F., and T.T. are involved in the larger research study of OR upon which the present study is based. In addition, H.N.F. and T.T. are responsible for introducing OR to Denmark and for translating and culturally adapting the program to a Danish context. NFL joined the project after the pilot study but is likewise involved in OR‐related research. In contrast, KN has no formal or informal affiliation with OR or related interventions.

This involvement of N.F.L., H.N.F., and T.T. in OR may be considered a potential limitation on one hand, but may also be viewed as providing a unique and in‐depth understanding of the phenomenon under investigation (Kvale and Brinkmann 2015). Regardless, the authors' professional engagement in OR required continuous reflexive attention throughout the research process. We sought to ensure that our preunderstandings were critically examined and allowed to be challenged, nuanced, and revised in light of participants' accounts. In the analytic process, particular care was taken to remain open to perspectives that did not align with our prior assumptions, including our findings that CAs can make relational responsibility visible and reveal potential gendered asymmetries.

Managing one's pre‐understandings is inherently complex, and from a hermeneutic standpoint, it is neither possible nor desirable to eliminate them entirely (Gadamer 2007). By maintaining explicit awareness of our pre‐understandings and by involving a co‐researcher who did not share these assumptions, we sought to adopt a critical and reflective stance throughout the research process.

### 2.4 Analytic Approach

The analysis was conducted using Longitudinal Interpretative Phenomenological Analysis, a longitudinal version of Interpretative Phenomenological Analysis (IPA; Smith et al. 2009). IPA is grounded in phenomenology, hermeneutics, and idiography, and seeks to provide an in‐depth, case‐oriented understanding of subjective meaning‐making (Smith and Osborn 2015). Its idiographic focus was well suited to examining how partners experience, interpret, and use CAs in their everyday lives.

The longitudinal approach enabled us to examine how participants interpreted their experiences and use of the CAs over time (Farr and Nizza 2019), attending to both continuity and change (Wanat et al. 2025). Consistent with IPA's idiographic commitment, analysis proceeded first at the individual participant level. Each participant's account was analyzed longitudinally across T1, T2, and T3. The analytic process involved close, line‐by‐line reading of transcripts, detailed initial noting, and the development of experiential themes grounded in participants’ own words and expressions. For each participant, particular attention was paid to how experiential themes developed, were maintained, or shifted across time. We conceptualized these developments as “ways” of experiencing, interpreting, and using CAs.

Following the individual‐level analyses, the two partners' accounts were brought together at the couple level. This analysis examined how partners' experiences, interpretations, and uses of CAs related to and informed one another, while retaining the distinctiveness of each partner's account. Finally, we conducted a cross‐couple analysis to identify shared and divergent ways of experiencing, interpreting, and using CAs across the six couples. This process enabled us to identify patterns across couples while preserving the idiographic detail of individual and couple trajectories.

The first author conducted the primary longitudinal analysis. The other authors contributed through review and discussion of transcripts, preliminary interpretations, and experiential themes. Selected material and emerging interpretations were also discussed with colleagues with expertise in qualitative and family research to critically examine and refine the analysis.

## 3 Results

Across the data, we identified three main ways in which couples experienced, interpreted, and used the CAs: (1) as instrumental contracts, (2) as expressions of mutual understanding, and (3) as making relational responsibility visible. These ways are not mutually exclusive. While some couples primarily aligned with one way, participants also emphasized other ways at different points in the data. In the following, we provide detailed accounts of a few illustrative examples to ensure analytical depth and richness, while additional examples are presented more briefly to highlight variation across participants and couples. In line with IPA recommendations, all couples are represented in the analysis, reflecting the advantage of a smaller sample in balancing depth and breadth (Smith and Osborn 2015).

### 3.1 Agreements as Instrumental Contracts: “I Just Haven't Been Able to Fulfill That Task”

The case of Kia and Carl's use of CAs about physical intimacy, time‐outs, and screen free time is an illustrative example of using the CAs in a rule‐governed and instrumental manner. Kia and Carl are a couple in their forties with three children, and both work full‐time. In the OR program they chose to work with two core concerns: Carl's primary concern was the nature of their relationship, which he described as a wish for them to “be more of a romantic couple rather than practical partners.” Kia's core concern related to their communication, particularly their recurrent pattern characterized by a mismatch between her intended meaning and Carl's interpretation. She experienced that Carl often construed her communication as personal criticism, despite her intention not being critical. They collaboratively decided on several CAs, among these were CAs about increasing physical intimacy, using time‐outs, and more screen free time together. Kia for instance agreed to express daily appreciation toward Carl through physical affection such as hugs and kisses.

In the first interview with Carl, he explicitly framed their CAs using concepts drawn from his work life, comparing them to Key Performance Indicators (KPIs)^1^:

… you could say it's almost like setting a KPI for yourself, right? And such a KPI must be measurable. And it's not just ones and zeros. There should be something quantitative to it, right? So that… that's how I think we work, you know?

(Carl, T2)

Besides KPIs, Carls also used other concepts from his work life when talking about their CAs concerning more physical intimacy, specifically hugs, kisses, and cuddling in the relationship. For instance, he emphasized that these CAs should be Specific, Measurable, Attractive, Realistic, and Time‐Bound (SMART),^2^ although these were not terms provided by the OR program:

[…] I have managers reporting to me (at work) […] If they don't make their goals specific enough or SMART enough, they won't succeed […] With my goal of more physical intimacy […] I have made sure that it is both realistic and achievable for both parties. I have had a dialogue about it so that my stakeholder—Kia—is involved in the entire goal. And I have set a timeline for myself, with a milestone that by July I should be able to sense a higher level of commitment and a feeling in Kia that says: “This is actually pretty okay and exciting”

(Carl, T2)

In this sense, hugs, kisses, and cuddling in their relationship were transformed into measurable entities, and the logic of optimization from his (and her) work life permeated his way of dealing with difficulties in their relationship. This logic sought to reduce complexity and instrumentalize physical expressions of affection such as hugs and kisses. In the second follow‐up interview, Carl continued to emphasize the importance of having CAs that were measurable, realistic, and specific, thus upholding the belief that changes in their relationship should be shaped by instrumental and optimization‐oriented logics:

I still think the comparison is relevant, because that's how we do things at work […] If you want something to change; “what do we need to change tomorrow in order to achieve something later on?”, then you must make it specific… You must have some milestones and so on.

(Carl, T3)

While this logic made the CAs meaningful and motivating for Carl, it had different consequences for Kia. In her first interview, Kia described how the CAs could be experienced as “contracts” and “rules”:

… we have an agreement… and at work I wouldn't just say; “Now I'll just do something completely different than we agreed”—I live up to the contract I've made—I think that's also what's at stake here; that I know this is what I've signed up for… These are the rules we have, the agreements […] It gives me a sense of safety. Because then I know what I have to live up to.

(Kia, T2)

While Kia initially experienced their specific and measurable CAs as tangible, safe, and helpful, this changed over time. In the follow‐up interview she described how failing to live up to the CAs intensified her feelings of guilt and inadequacy:

I think it went well at first, and we were both good at sticking to the agreements we'd made. And then… yeah… It just really hasn't been that way for the past many months. I must admit, I haven't… I mean, the agreement we had, that I should show him more physical affection, and the whole thing about nurturing our romantic relationship… I just haven't been able to fulfill that task […] I just feel guilty about it all the time […] I just feel inadequate, you know.

(Kia, T3)

Thus, Kia's experience of their CAs changed over time. The contractual way of using the CAs initially made them feel tangible; however, over time, the rule‐based nature of the CAs intensified Kia's sense of guilt and inadequacy. It is important to note that, for Kia and Carl, the rule‐based CAs did not only concern kisses and hugs but also, for example, screen free time in the evening before going to bed in order to increase their time together. Kia also expressed feeling guilty about not fulfilling this CA, as well as their CA regarding kisses and hugs, suggesting that the specific content of the CAs may be less important than how they are experienced, interpreted, and used in everyday life.

Kia was not the only participant who expressed negative emotions related to not fulfilling CAs when they were viewed as rules to be followed. Sarah also experienced negative emotions when she was unable to succeed with her rule‐based CAs. Sarah and Michael are a couple in their forties with two children, both working full‐time. They chose to work on a core concern in the OR, which they described in the program as a “trust issue.”

Sarah and Michael developed several CAs. For example, Sarah committed to giving Michael an advance notice when she wanted to discuss sensitive or relationship‐related concerns, as he found such conversations particularly difficult. She also agreed to use time‐outs when needed, to feel less hesitant about asking Michael questions, and to be more attentive and receptive when he shared his thoughts and experiences. Michael's CAs focused on being “more transparent about what I am doing, who I am doing it with, and when.” In the interview with Sarah, she described how she attempted to use some of the CAs in their everyday life without success, and how this left her feeling sad and disappointed:

I tried to have a conversation with him after the program ended. I used some of those techniques we agreed on: both inviting him into the conversation, letting him decide when it should happen, telling him what my purpose with the conversation was, and so on. And still, he just went: “Oh no!” I mean, we weren't very far into the conversation before he became distant and cold… like a wall you just slide off, you know? And I just… wasn't met at all, not at all […] I should have used one of the next techniques we had planned: the thing about stopping the conversation and simply saying: “time‐out”. But I didn't do it… I mean, I just fell into one of those emotional holes and became so sad and disappointed.

(Sarah, T2)

Sarah seems to understand the CAs as a set of rules to follow, and when she is unable to follow these rule‐based CAs successfully—and when the effect of using them does not elicit the expected response from her partner—she expresses sadness and disappointment.

In addition to increasing feelings of sadness and disappointment for some couples, the CAs may also intensify existing polarization between partners. Maya and Fred's use of their CAs about communication strategies can exemplify this problem. They are a couple in their thirties with three children, both working full‐time. In the program, they chose to focus on a core concern they called “angry communication.” Maya and Fred developed several CAs, mostly concerning specific communication strategies, such as using time‐outs, applying the speaker–listener technique, and maintaining a neutral tone of voice during conflicts.

In the interview, Maya described how the CAs ‐ and not living up to them ‐ sometimes fueled conflicts and increased polarization between her and her partner, Fred. She explained: “…it just becomes yet another way he can accuse me” (Maya, T3). This suggests another variation of instrumental use of CAs, in which they become fuel for pre‐existing conflicts within the relationship, especially when partners are unable to live up to or follow such agreements. Thus, CAs can be experienced, interpreted, and used as rules to be followed, giving rise to unintended consequences, such as increased feelings of guilt, sadness, or polarization within the relationship.

#### 3.1.1 Consequences of Agreements as Instrumental Contracts

When CAs are framed through instrumental and optimization‐oriented logics, the meaning of the CA can shift into evaluative benchmark against which individual partners evaluate themselves. When affection, screen free time, and communication techniques become something to deliver in accordance with a contract, relational dynamics risk being reorganized around performance and compliance. The CA establishes a standard, and with it, the possibility of falling short. CAs can thereby intensify, rather than alleviate existing feelings of guilt. When CAs are understood as contracts that need to be fulfilled, relational difficulties risk becoming individualized and experienced as personal failure.

Moreover, optimization logics tend to reduce relational complexity. By isolating specific behaviors as targets for improvement, they risk overlooking the affective, relational, and contextual conditions that make such behaviors meaningful. Affection, screen free time, and communication can then become embedded in obligation, rather than authentic expressions of attention, understanding, or affection. While this may create short‐term behavioral change in some cases, it can also introduce pressure that undermines the very intimacy it seeks to enhance. Thus, instrumental ways of using CAs may intensify critical self‐evaluation and polarization between partners.

### 3.2 Agreements as Mutual Understanding: “We Have a Completely Different Level of Awareness”

For some couples, the CAs were closely associated with a mutual understanding of what was important or valuable to the other person and the relationship. In this way, the CAs provided a means of enacting this shared understanding. As a result, couples interpreted and used the CAs more flexibly, rather than as rules to be strictly followed, and the emphasis was not on specific behaviors but on an awareness of the other person's fundamental values, wounds, and wants. Dan and Sophie's way of using their CA about time‐outs exemplify this. Dan and Sophie are a couple who have one child, and both work full‐time. In the OR, they chose to focus on a core concern they described as “a lack of communication leading to misunderstandings and conflicts.” They developed several CAs, for example taking time‐outs during conflicts. For Dan and Sophie, these CAs were less a rule to be followed than a way of expressing a new understanding of each other's needs. Dan explained:

… If Sophie needed to have 10 minutes, then previously I would have been the type who just said: “No, now!” And that doesn't help… But now, because we have talked about it, then it's maybe a bit more acceptable. So; “Yes, take your 10 minutes, but we also need to get it resolved…” […] Having talked things through means that we have a completely different level of *awareness* about the other person […] that's the big turning point for me.

(Dan, T2)

Dan emphasized that it was the awareness about his partner that had changed. He stated that it was not the agreements themselves, but their connection to a greater mutual understanding that made new ways of acting and relating possible for them. Dan's partner, Sophie, emphasized a similar experience with the CAs:

Interviewer: Can you think of a specific situation where you, or Dan, have used that agreement?

Sophie: Not consciously, I don't think. I mean, it's more something that sort of sits a bit in the subconscious in some way, or it's like, “a seed has been planted” if you can put it that way? And you're somewhat aware of it, but not in the sense that I would literally take out a tool and say, “just remember this rule.”

(Sophie, T3)

As Sophie pointed out, their mutual understanding was not grounded in a rigid, rule‐based logic and did not prescribe every action; rather, it oriented her attention toward what they had prioritized together. This way of using the CAs was supported by Dan: “I mean, I do think it's nice enough that we have them. But it's not like we ever really need to enforce them and say: “remember, we have this agreement and so on.” (Dan, T3).

The importance of grounding the CAs closely in a mutual understanding is also key for the ways in which Annie and James used their CAs about planning‐related matters. Annie and James are a couple with two children, both working full‐time. In the OR, they chose to focus on a core concern which they described as “losing connection as a couple and feeling alone within the relationship.” Annie's core concern was feeling less alone with the practicalities and the mental load in managing the demands of family life. James generally struggled with maintaining an overview of everyday tasks, which made it difficult for him to keep track of the practical day‐to‐day tasks at home. At the same time, he wanted to support Annie so she could have more “mental space,” and so that they could both have more time and energy to “simply enjoy being a couple.”

Annie and James made a CA to purchase a whiteboard and agreed that whenever he asked her about practical or planning‐related matters (e.g., what they were having for dinner), she could simply respond by saying, “check the board” instead of having to carry all such information herself. Annie explained that since they made this agreement, James no longer became annoyed when she did not provide an immediate answer, and she no longer felt frustrated by his questions about everyday practicalities:

Interviewer: What do you think is different now, since he doesn't get upset?

Annie: I think it's because we've talked about it. We agreed that it was okay for me to say that […] He understood that it is so I don't have to carry it in my head… and go around expecting him to ask about it. Because we'd agreed that he should just go look himself.

(Annie, T2)

This illustrates how CAs can work primarily as a materialization of a mutual understanding between partners and function as a means of navigating and establishing common ground in relation to managing potential conflict situations in everyday life. For Annie and James, the use of a whiteboard and their associated understanding of each other increased their shared responsibility for household practicalities over time. As illustrated above, Annie initially used the whiteboard as something she could point to, while by the second follow‐up interview James had become the partner responsible for writing dinner plans on the board and visualizing their plans and to‐dos. In this sense, CAs can be used as materializations of a mutual understanding developed, serving as a way of enacting this understanding in the couple's everyday life.

#### 3.2.1 Consequences of Agreements as Mutual Understanding

When CAs are experienced, interpreted, and used as expressions of mutual understanding, their consequences are less about behavioral compliance and more about relational reorientation, attunement, and understanding. Rather than operating as contracts or rules to be enforced, the CAs become shared orientations that reshape how partners interpret each other's actions.

Importantly, CAs grounded in mutual understanding can also evolve over time. As illustrated in Annie and James' use of the whiteboard, what began as a symbolic marker of awareness gradually redistributed practical and mental responsibility. In this way, CAs can not only reflect a new understanding but also materialize and extend it over time. Their consequences can therefore be cumulative; they can consolidate new meanings, scaffold new practices, and embed a sense of shared orientation into the fabric of everyday life.

### 3.3 Agreements Can Make Relational Responsibility Visible: “Are There One or Two of Us Who Are Invested in This?”

For some couples, the use of CAs in their everyday lives revealed an uneven distribution of relational responsibility between partners. Jane and John's way of using their CAs about communication strategies is an example of this. Jane and John are a couple in their forties with one child. He works full‐time, while she was unemployed during the study period. In the OR, they chose to work on one core concern, which they defined as “a lack of alignment in their communication.” As part of the OR, they formulated several CAs related to this concern. Jane committed to respect John's need for time‐outs and to communicate in “a more concise and precise manner.” John, in turn, committed to show greater patience when listening to her, accepting changes in plans, and returning to the conversation after a time‐out in order to re‐engage in communication.

In an interview with Jane, she described how she experienced herself as bearing the sole responsibility for upholding their CAs, while her partner, John, devoted most of his time and energy to his work life:

Jane: …it's also me who must be like: “We had an agreement, didn't we?” […] and then it just fizzles out. So, it doesn't work if the agreement isn't actually an agreement. So, I feel a bit like I must be the one leading everything. And then it doesn't work, I don't think, if it isn't shared responsibility between us […] Then I feel a bit like: are there one or two of us who are invested in this?

Interviewer: And when you start to think that what kind of feeling do you get?

Jane: Well, a kind of uncertainty about our foundation, really.

(Jane, T2)

Experiencing primary responsibility for implementing the CAs left Jane feeling alone and uncertain about their level of mutual commitment to the relationship. Her partner, John, also reflected on their CA about taking time‐out during conflict. In his first interview, he explained that he had not used the agreement, stating that; “there has been no time for it […] I've just been very busy at work” (John, T2). From John's perspective, the uneven responsibility associated with the CAs is largely explained by differences in their work lives—he is busy with his job while Jane is not currently working—but also by what he perceived as their differing conversational needs. As he put it, “when Jane used to go to work, where she talked *all* day, her quota of talking was never used up anyway.” (John, T2).

For Jane, however, the uneven responsibility took on a markedly different meaning. Rather than reflecting practical differences in everyday circumstances or differing conversational needs, she interpreted John's lack of engagement with the CAs as a sign that she was left alone carrying the emotional and relational work of the relationship, as well as an indication of his limited commitment. Jane was not the only one who described the experience of an uneven distribution of responsibility in implementing the CAs in the couple's everyday lives. Sarah similarly explained that the responsibility for carrying out the changes they had agreed upon during the OR ultimately fell to her:

…we can't manage to sit down and construct that space. I mean… [long pause] and then it also becomes difficult because it's very clear that Michael feels it has been hard to participate in […] when he has difficulty figuring out what he feels and putting it into words, then he must make more of an effort to be in this than I do. And therefore, it also ends up being me who must hold on and take responsibility, if it has to be in this structured way.

(Sarah, T2)

This is particularly interesting because Sarah and Michael addressed the question of responsibility for maintaining and implementing the CAs during their fifth coach session. At that point, Michael stated that “I am pretty sure that I can do these changes […] I don't think it's her responsibility” (Michael, T1), and Sarah similarly expressed that she imagined it would be “moderately easy” (Sarah, T1) to implement the CAs in their everyday lives. This suggests a mismatch between couples' initial expectations of how easily they would be able to implement their CAs in everyday life and the greater difficulties they later experienced when trying to carry them out.

Furthermore, from Sarah's perspective the uneven distribution of relational responsibility was closely connected to Michael's difficulties in identifying and articulating his emotions. Michael himself partly agreed with this interpretation. When asked about the CAs in the first interview, he explained:

We probably haven't really followed them. I don't think we have […] Well, we haven't really talked about our relationship… not since the program ended actually […] it's associated with a certain nervousness for me to start doing that.

(Michael, T2)

This illustrates how attempts to implement specific or structured CAs in everyday life may bring underlying relational asymmetries into view, as partners differ in their capacities, motivations, needs, and emotional readiness to engage with the practices that the CAs presuppose.

#### 3.3.1 Consequences of Agreements Making Relational Responsibility Visible

When CAs make relational responsibility visible, their consequences may illuminate asymmetries in the relationship. In such cases, the attempt to implement the CAs reveals who carries the primary responsibility for sustaining the relationship in everyday life. As Jane's reflections suggest, carrying the primary responsibility for upholding the CAs can raise doubts about whether commitment to the relationship is truly shared: “are there one or two of us who are invested in this?” The consequence is therefore not only momentary frustration, but a more fundamental uncertainty about the foundation of the relationship itself.

At the same time, the disclosure of uneven responsibility may shape how partners experience their CAs. As the example of Sarah illustrates, the techniques themselves do not simply “fail.” Rather, the difficulty of enacting them makes it visible that she is the one preparing, initiating, structuring, and attempting couple conversations as well as repairs. This visibility may intensify negative emotions such as sadness and disappointment—not only because the intended conversation fails, but because the responsibility for making it succeed appears to rest disproportionately on one partner.

Importantly, the uneven responsibility that becomes visible through the CAs can be interpreted differently by the partners involved. For example, John explained the imbalance primarily in terms of practical differences in their everyday lives and communication needs, whereas Jane experienced it as a sense of being alone in maintaining the relationship and as a lack of mutual commitment. These differing interpretations illustrate how the meaning of relational responsibility is negotiated within the couple and embedded in broader interpretations of everyday life circumstances and emotional capacities.

CAs that were initially intended as mutual commitments may thus become additional relational tasks to manage. When one partner struggles to engage—whether due to competing priorities, discomfort, or difficulties articulating emotions—the other may feel compelled to compensate. In this way, CAs can amplify imbalances in mental and emotional load already present in the relationship. Notably, in both cases the partner who experienced carrying the primary responsibility for implementing, initiating, and sustaining the CAs was the female partner.

Importantly, these consequences do not necessarily stem from the CAs themselves, but from the relational dynamics into which they are introduced. When there is not a relatively symmetrical willingness or capacity to engage, the CAs may render existing inequalities more explicit. In this sense, the CAs make visible who initiates, who withdraws, and who carries the responsibility for repair. Such visibility may either open possibilities for renegotiating relational responsibility or intensify experiences of isolation and imbalance within the relationship.

## 4 Discussion

The present study is a qualitative longitudinal study examining how couples experienced, interpreted, and used the CAs they developed in the online OR program, within their everyday lives. Across the data, we identified three different main ways in which couples experienced, interpreted, and used the agreements: (1) as instrumental contracts, (2) as expressions of mutual understanding, and (3) as making relational responsibility visible. Together, these ways illustrate that the meanings and consequences of CAs are both embedded into the practices of everyday life and may change considerably over time. In some cases, CAs that were initially experienced as helpful later became evaluative benchmarks against which partners assessed their own and each other's performances as successes or failures. In other cases, CAs that began as symbolic expressions of shared understanding gradually evolved into embedded everyday practices, as illustrated by Annie and James’ use of a whiteboard to coordinate household responsibilities. These changes over time illustrate that the meanings and consequences of intervention elements (such as specific CAs for couples) may not be fully understood when they are formulated and negotiated as part of an intervention. Rather, their meanings and consequences continue to evolve as they encounter the material, emotional, and relational complexities of everyday life over time.

These results have implications for how change processes are conceptualized within couple interventions. Taking a critical psychological perspective made it possible for us to see how CAs are not simply tools for promoting desired behaviors but become embedded into the ways in which couples coordinate, organize, and negotiate their everyday lives. For example, several participants reframed CAs using language from their work life, demonstrating how other social practices can permeate intimate relationships. By focusing on the conduct of everyday life, the analysis highlights the contextually embedded nature of relational change and makes it visible how participants actively transform intervention elements in accordance with the concerns and demands of their everyday lives. Thus, relational change can be understood not simply as adherence to behavioral rules (Jacobson and Margolin 1979) or as the gradual emergence of new interaction patterns through partners' ongoing responses to one another (Christensen et al. 2020), but as a process through which intervention elements acquire meaning as they are continuously experienced, interpreted, and used within the social practices that constitute couples' everyday lives. This perspective extends existing understandings of change in couple interventions by shifting attention from *what* couples are encouraged to change toward *how* change efforts become part of couples' conduct of everyday life.

The results also have implications for the design and implementation of online couple interventions. Supporting couples in formulating realistic, meaningful, and tangible CAs remains important. However, the present findings suggest that this alone may be insufficient. Intervention design may also benefit from helping couples reflect on how CAs may be experienced, interpreted, and used once the program ends, and how different ways of engaging with CAs may have different consequences for partners and the relationship as a whole. This awareness could be incorporated in several ways. For example, online interventions could include case examples, video demonstrations, or reflective exercises demonstrating different ways couples may experience, interpret, and use CAs in everyday life. Such program content may help couples recognize both flexible and more rigid ways of engaging with CAs and support ongoing adaptation and adjustment over time. Similarly, booster sessions, follow‐up contacts, or integration with online or traditional couple therapy may provide opportunities to revisit not only the content of CAs but also how they are experienced, interpreted, and used within the context of everyday life over time.

The present findings also invite consideration of the role of CA content. Although the primary focus of this study has been on how CAs are experienced, interpreted, and used in everyday life, questions remain regarding whether some domains may be particularly sensitive to the ways CAs are subsequently understood and enacted. This may be especially relevant in areas such as physical intimacy, affection, and sexuality, which previous research has identified as both highly important to many couples and often under‐addressed in couple therapy (Eldridge et al. 2022).

On the one hand, allowing couples to formulate CAs concerning intimacy may represent a particular strength of interventions such as OR. The structured format provides opportunities to articulate wishes, concerns, and needs that might otherwise remain unspoken. For some couples, discussing such issues within a self‐help intervention may feel less stigmatizing and more accessible than addressing them in traditional couple therapy (Hubbard and Harris 2020; Hubbard and Anderson 2022). Moreover, the process of formulating and discussing CAs may itself help partners develop a deeper understanding and possibly acceptance of each other's wishes, needs, and intentions.

On the other hand, intimacy‐related CAs may be especially vulnerable to instrumental, evaluative, or rule‐governed ways of use. When affection, sexuality, or physical intimacy become translated into measurable goals or obligations, CAs may be experienced as performance demands rather than as expressions of genuine care and connection. Importantly, however, the present findings suggest that these challenges arise not primarily because of what the CAs are about, but because of how they are experienced, interpreted, and used in everyday life. Similar dynamics emerged across a range of different CAs, including communication strategies, screen free time, and efforts to increase time spent together. Thus, while some domains may be particularly sensitive, difficulties appear to be closely tied to how CAs become integrated into couples' everyday practices. This suggests that intervention design should focus not only on helping couples formulate CAs, but also on supporting flexible, reflective, and meaning‐oriented ways of using them.

Another important point for discussion concerns the potential gendered distribution of relational responsibility. In the cases presented here, the responsibility for initiating and sustaining the practices associated with the CAs appeared to fall primarily on the female partners. While the present study cannot draw broader conclusions about gendered patterns, this finding resonates with previous research suggesting that women often assume a disproportionate share of the emotional (McQueen 2023), cognitive (Daminger 2019), and relational work involved in maintaining couple relationships in heterosexual relationships (Doucet 2018; Duncombe and Marsden 1995; Twamley and Faircloth 2024).

If such gendered dynamics remain unaddressed, unequal responsibility for maintaining the relationship may be understood as a matter of individual motivation, need, competence, or commitment rather than as a relational, cultural, and structural pattern (Haavind 1994; Lindvåg and Thoresen 1994; Smoliak et al. 2025). Making gendered patterns of relational responsibility visible is not only analytically important but may also provide partners—particularly those who carry a disproportionate share of relational work—with new ways of understanding the implicit expectations and pressures surrounding care and responsibility within romantic relationship in contemporary Western society. Such understandings may, in turn, open up possibilities for renegotiating everyday practices and thereby contribute to relational change.

Focus on gendered patterns of relational responsibility could be implemented in different ways. Couples and coaches could, for example, be introduced to research‐based knowledge on gendered patterns inspired by this study, as well as by work in the field of contemporary parenting culture (Faircloth 2013; Lee et al. 2023; Twamley and Faircloth 2024). Notably, the couples in this study were all parenting couples who navigate the everyday constraints of combining partnering and parenting. As Faircloth (2020) shows, parenting couples are faced with highly gendered cultural, material, and physical barriers to the ideals of egalitarian romantic couple relationships.

Additionally, previous research suggests that online couple interventions may create opportunities for initially less‐motivated (male) partners to become more engaged in addressing relationship difficulties (Lassen et al. 2025). Online interventions may therefore simultaneously interact with and potentially reshape existing patterns of relational responsibility and engagement. On the one hand, intervention practices that require partners to initiate conversations, monitor progress, or sustain reflective dialog may risk placing additional responsibility on the partner who is already oriented toward relational care. On the other hand, the structured format of online programs may also create opportunities for partners who are less accustomed to engaging in relational dialog to participate in new ways.

For practitioners and program designers, these findings highlight the importance of attending to how responsibility for engaging with intervention tasks is distributed within the couple. Explicitly addressing how partners share responsibility for initiating and sustaining relational conversations may help prevent intervention practices from inadvertently reinforcing existing asymmetries while supporting both partners in taking active roles in relational change.

In summary, the study findings point to the importance of maintaining a dual focus in both research and practice: attending to the content of intervention elements while simultaneously examining the processes through which they are experienced, interpreted, and used in everyday life. Without such a social practice‐oriented perspective, there is a risk that interventions overemphasize *what* couples should change while underestimating the significance of *how* change efforts are experienced, interpreted, and used within the couples' everyday lives. Attending to these processes may strengthen both theoretical understandings of relational change and the practical relevance of online couple interventions.

### 4.1 Study Limitations and Strengths

Some study limitations should be noted. First, the study reflects a deliberate methodological prioritization of depth over breadth. By focusing on a small number of cases and examining them in detail across multiple time points, the analysis provides rich insight into how CAs are experienced, interpreted, and used by couples in their everyday lives. However, the small and relatively homogeneous sample limits the extent to which variations across a broader range of couples and social contexts can be captured. All participants were White/Caucasian different‐sex co‐parent couples who had completed compulsory schooling, and 11 of 12 participants were employed. Future research could build on these findings by examining how different ways of using CAs relate to longer‐term relational outcomes across larger and more diverse samples, including couples from more socioeconomically disadvantaged backgrounds and diverse racial, ethnic, or family backgrounds.

Second, it is important to note that N.F.L., H.N.F., and T.T. are involved in the broader implementation and study of the OR program in Denmark, and that H.N.F. and T.T. have played a central role in introducing IBCT and OR in Denmark. While this involvement has provided valuable contextual knowledge and access, it may also have shaped our pre‐understandings. Efforts were made throughout the research process to maintain reflexivity and transparency in relation to these positions.

Alongside these limitations, this study has several important strengths. One of the strengths of the longitudinal qualitative approach is its capacity to bring unanticipated perspectives into view. Throughout the analytic process, particular care was taken to remain open to perspectives that did not align with our prior assumptions. This was exemplified by the finding of the potential gendered distribution of responsibility for initiating and sustaining CAs.

Furthermore, the longitudinal design and repeated interviews with both partners made it possible to examine how couples' experiences, interpretations, and use of CAs evolved over time, rather than relying solely on retrospective accounts from a single time point. In addition, coach session transcripts provided insight into how CAs were initially formulated and discussed during the intervention. Finally, by focusing on a single intervention element rather than the program as a whole, the study contributes to a more detailed understanding of how intervention elements become part of couples' conduct of everyday life.

## 5 Conclusion

This study shows that CAs do not transfer directly from intervention to everyday life. Instead, they are continuously interpreted, negotiated, and transformed within couples' relational practices. The three identified ways of experiencing, interpreting, and using the CAs—as instrumental contracts, mutual understanding, and making relational responsibility visible—demonstrate that their meanings and consequences vary over time and across contexts. These findings highlight the importance of complementing a focus on intervention content with attention to how these are integrated in everyday life. Supporting couples in reflecting on how CAs are experienced, interpreted, and used may be central to fostering meaningful and sustainable relational change.

## Ethics Statement

Following OR, participants were invited to participate in the present qualitative study and provided informed consent. Quotations are presented using pseudonyms, with identifying details omitted or altered. Participants were not compensated for the interviews. The study has been approved by the Research Ethics Committee of Aarhus University (Project ID BSS‐2024‐018‐S2).

## Data Availability

Data that support the findings of this study are available on request from the corresponding author. The data are not publicly available due to privacy or ethical restrictions.

## Footnotes

1 A Key Performance Indicator (KPI) is a quantifiable, measurable value that organizations use to track and evaluate their progress toward achieving specific strategic, financial, and operational goals.

2 SMART is an acronym that stands for Specific, Measurable, Attractive, Realistic, and Time‐Bound. A SMART goal incorporates all these criteria. SMART goals are used in different settings, most commonly in work settings.
